# The Application of Vibrational Spectroscopy Techniques in the Qualitative Assessment of Material Traded as Ginseng

**DOI:** 10.3390/molecules21040472

**Published:** 2016-04-12

**Authors:** Maxleene Sandasi, Ilze Vermaak, Weiyang Chen, Alvaro Viljoen

**Affiliations:** 1 Department of Pharmaceutical Sciences, Faculty of Science, Tshwane University of Technology, Private Bag X680, Pretoria 0001, South Africa; sandasim@tut.ac.za (M.S.); vermaaki@tut.ac.za (I.V.); chenw@tut.ac.za (W.C.); 2 SAMRC Herbal Drugs Research Unit, Faculty of Science, Tshwane University of Technology, Private Bag X680, Pretoria 0001, South Africa

**Keywords:** ginseng, *Eleutherococcus senticosus*, *Panax ginseng*, *Panax pseudoginseng*, *Panax quinquefolius*, hyperspectral imaging, mid-infrared spectroscopy, near infrared spectroscopy, UHPLC-MS

## Abstract

The name “ginseng” is collectively used to describe several plant species, including *Panax ginseng* (Asian/Oriental ginseng), *P. quinquefolius* (American ginseng), *P. pseudoginseng* (Pseudoginseng) and *Eleutherococcus senticosus* (Siberian ginseng), each with different applications in traditional medicine practices. The use of a generic name may lead to the interchangeable use or substitution of raw materials which poses quality control challenges. Quality control methods such as vibrational spectroscopy-based techniques are here proposed as fast, non-destructive methods for the distinction of four ginseng species and the identification of raw materials in commercial ginseng products. Certified ginseng reference material and commercial products were analysed using hyperspectral imaging (HSI), mid-infrared (MIR) and near-infrared (NIR) spectroscopy. Principal component analysis (PCA) and (orthogonal) partial least squares discriminant analysis models (OPLS-DA) were developed using multivariate analysis software. UHPLC-MS was used to analyse methanol extracts of the reference raw materials and commercial products. The holistic analysis of ginseng raw materials revealed distinct chemical differences using HSI, MIR and NIR. For all methods, *Eleutherococcus* *senticosus* displayed the greatest variation from the three *Panax* species that displayed closer chemical similarity. Good discrimination models with high R^2^X and Q^2^ cum vales were developed. These models predicted that the majority of products contained either /*P. ginseng* or *P. quinquefolius*. Vibrational spectroscopy and HSI techniques in tandem with multivariate data analysis tools provide useful alternative methods in the authentication of ginseng raw materials and commercial products in a fast, easy, cost-effective and non-destructive manner.

## 1. Introduction

Ginseng, an ancient herbal medicine with a long history of traditional use, is one of the most popular and best-selling herbal medicines in the world [1,2]. The word ginseng, literally meaning “man root”, refers to the characteristic shape of the roots which resemble the human body and the legs of a man [3]. Traditionally, the term ginseng was applied to preparations formulated using *Panax* species, but nowadays it refers to any product said to have adaptogenic or restorative properties [2]. This gives rise to quality control problems due to botanical misidentification. The word “ginseng” refers to *Panax ginseng* C.A.Mey. (Asian/Oriental ginseng—Korean and Chinese), *P. quinquefolius* L. (American ginseng) and *Eleutherococcus senticosus* (Rupr. & Maxim.) Maxim. (Siberian ginseng) amongst others, all belonging to the Araliaceae family. The roots of these plants are considered to be very valuable, especially in Traditional Chinese Medicine. The processed form of Asian ginseng is used to replenish “vital energy” as it is “warm”, while American ginseng is used for reducing “internal heat” as it is “cool” [4]. *Panax* species are used worldwide for their anti-stress, CNS-stimulating, anticancer, antidiabetic and antihypertensive activity, in addition to improving physical performance and sexual function [1,4]. *Eleutherococcus* (or *Acanthopanax*) *senticosus* is known as an adaptogen and in Traditional Chinese Medicine it is used to invigorate qi, nourish the kidney and strengthen the spleen [5]. The *Panax* species all contain ginsenosides which are unique to the species and the relative concentrations can be used in certain cases to differentiate between species. *Eleutherocossus senticosus* on the other hand is chemically distinct from *Panax* species in that it contains eleutherosides [2].

The root material of the various ginseng species are similar in appearance, especially when provided in powder form or slices, which makes species identification more challenging [4]. The commercial importance also has to be considered as the monetary value of American ginseng was deemed to be higher than that of sun-dried Asian ginseng. It is evident that authentication of the ginseng raw materials is paramount to ensure that the correct botanical source is used [4]. Numerous methods have been developed to identify and quantify ginsenosides in different ginseng species and products including thin layer chromatography (TLC), high performance liquid chromatography coupled to ultraviolet detection (HPLC-UV) and liquid chromatography coupled to mass spectrometry (LC-MS) [6]. HPLC coupled with evaporative light scattering detection (HPLC-ELSD) was used to simultaneously quantify 14 major ginsenosides as marker compounds for *Panax ginseng*, including white and red (processed) ginseng [6]. In another study, an LC-MS method was developed to authenticate raw material and commercial products of *P. ginseng* and *P. quinquefolius* [4]. HPLC and HPLC-tandem mass spectrometry (HPLC-MS) were also used to identify and quantify seven ginsenosides and two eleutherosides in twenty-five commercial ginseng products [2]. It was found that in the cohort of US ginseng products tested, the plant species of all the products were correctly identified. They reported significant product-to-product variability and deviations from the labeled concentrations of marker compounds. Ginseng products contain diverse compounds and most quality control methods are limited to a small range of marker molecules. Thus, holistic approaches such as two-dimensional J-resolved proton NMR spectroscopy and multivariate data analysis have been investigated to discriminate different ginseng preparations using metabolic fingerprinting [7].

As the chromatographic analysis of raw material requires skilled personnel, is costly and labourious, the past few years have also seen the introduction of new, simple, rapid quality control techniques like vibrational spectroscopy. The most commonly used vibrational spectroscopy techniques include near infrared (NIR) and mid-infrared (MIR) spectroscopy. These techniques have been successfully applied in the quality control of pharmaceutical, petrochemical, agricultural and food products as well as herbal medicines [8]. They offer advantages such as being simple, rapid, non-destructive and chemical-free with no sample preparation required. Samples can be measured in different physical states *i.e.*, solids, liquids, pastes and gases [9]. Conventional spectroscopic methods cannot measure the internal constituent gradients within a sample, since spatial information cannot be captured. To address this limitation that results in discrepancies between measured and predicted compositions, hyperspectral imaging, which combines conventional spectroscopy and digital imaging, was developed [10]. Hyperspectral imaging (HSI) is a technique typically used in aerospace and defense applications to visualise the surfaces and composition of objects, but also in pharmaceutical analysis in the identification and compositional analysis of pharmaceuticals or in the detection of contaminants. This study aims to develop fast, non-destructive protocols for the distinction of ginseng root material and the identification of botanical species in commercial ginseng products using spectroscopy-based techniques (including HSI, MIR and NIR) in combination with multivariate data analysis techniques.

## 2. Results and Discussion

### 2.1. Qualitative Differentiation of Ginseng Raw Materials Using HSI Spectroscopy

**Figure 1:**
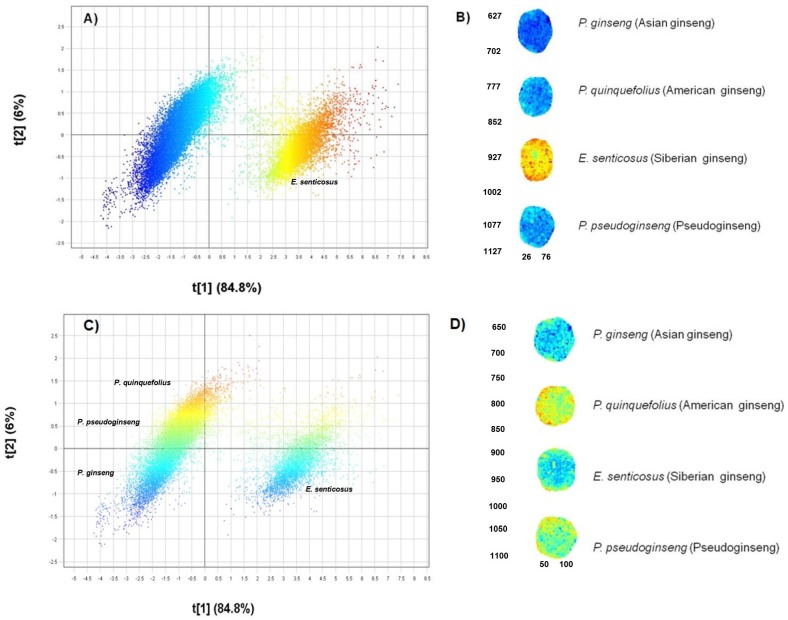
(**A**) Scatter 2D image (t1 *vs*. t2) showing pixel separation and coloured according to t[1] score value; (**B**) Corresponding score image of t[1]; (**C**) pixel clusters coloured according to t[2] score value; (**D**) corresponding score image of t[2].

The use of HSI to determine chemical variation between the different ginseng species yielded the results presented in Figure 1A–D.

**Figure 2:**
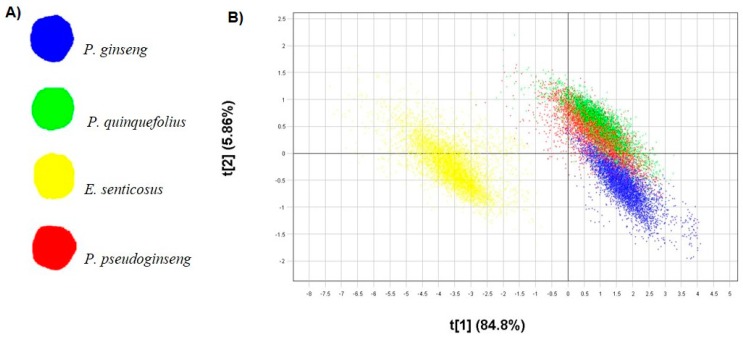
(**A**) PLS-DA Y-image of ginseng root powders colour-coded according to the species and (**B**) the corresponding pixel plot (PLS1 *vs.* PLS2) coloured according to the score values which correspond to the species in the score image.

The PCA model demonstrated good calibration statistics following mean centering and standard normal variate (SNV) pre-treatment of the data set. Three principal components (PCs) were used to model 95.3% variance in the data with 90.8% variation explained along PC1 and PC2. The different PC combinations were assessed in the score plot and the combination that displayed the greatest variance, *i.e.,* PC1 *vs.* PC2, was selected. The score scatter plot (Figure 1A) shows two distinct clusters separated along PC1 with a variation of 84.8%. When coloured according to the t[1] (PC1) score value which corresponds to the score image (Figure 1B), it is clear that *E. senticosus* (red-yellow) is chemically distinct from the other *Panax* species (blue) which are represented by lower score values (blue) in the score image (Figure 1B). The pixel scatter plot thus clearly demonstrates chemical segregation of *E. senticosus* (PC1+) pixels from the pixel cluster representing the three *Panax* species (PC1−). Evaluation of PC2 displayed minimal chemical variation between the three *Panax* species where only 6% variation was observed. Figure 1C shows the pixel scatter plot coloured according to the t[2] (PC2) score value which identifies *P. quinquefolius* pixels (orange-yellow cluster) on PC2+, *P. pseudoginseng* (yellow-green cluster) overlapping with *P. quinquefolius* while *P. ginseng* (blue cluster) occupies PC2−. The small variation observed in the scatter plot can also be seen in the score image where *P. quinquefolius* powder has an orange-yellow score value in the t[2] image compared to other species (Figure 1D). Variation modelled along PC3 did not further differentiate the three species significantly. Following PCA, PLS-DA classification modelling was performed and Figure 2A shows the PLS-DA Y-image colour-coded according to the species. The corresponding pixel scatter plot is shown in Figure 2B demonstrating the chemical distinction of *E. senticosus*. Separation of the three *Panax* species along PLS factor 2 is minimal (5.9%) between *P. ginseng* (blue), *P. quinquefolius* (green) and *P. pseudoginseng* (red) as observed in the pixel scatter plot. Pixel cluster overlap between *P. quinquefolius* (green) and *P. pseudoginseng* (red) is observed demonstrating that the two species are chemically more similar. The use of the HSI technique has not only proven useful for differentiation of plant species misidentified due to morphological similarities [11,12] but differentiation of fungal [13] and algae species [14] has also been achieved. The developed PLS-DA model was then used to predict a test set that comprised of authentic reference samples and commercial products to identify raw material constituents present in commercial ginseng products.

**Figure 3:**
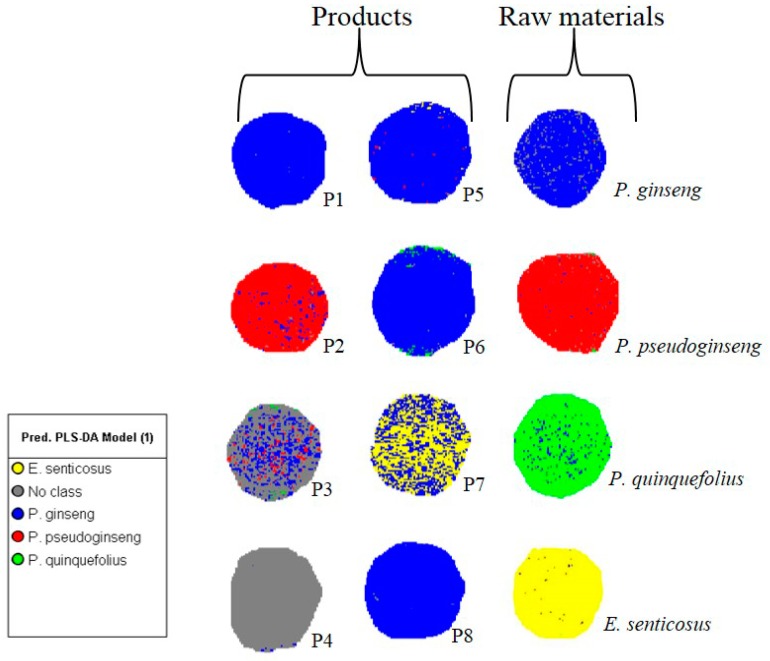
PLS-DA predictions for commercial products and authentic reference materials (test set) based on hyperspectral imaging data.

Figure 3 shows the prediction image (test set) consisting of known reference material and commercial products (P1–P8) coloured according to the predicted classes. The predictive ability of the model in classifying new samples was confirmed as the predicted species for reference materials matched the known identity. Additionally, the high Q^2^ value (0.78) also demonstrated that the PLS-DA model would accurately predict the raw material constituents of commercial products. Prediction of the commercial products classified four (P1, P5, P6, P8) of the eight products as containing *P. ginseng*, one (P2) containing *P. pseudoginseng*, two samples comprised of more than one species (P3 and P7) and for one sample (P4) the ingredients could not be matched to any of the ginseng reference materials used for calibration (Figure 3). Table 1 provides a summary of the prediction results with the percentage pixel abundance corresponding to each predicted species for the commercial samples. In the present study, it was possible to detect different ginseng species present in commercial products from scanning the products and introducing these into a robust and well calibrated model. Quality control aspects that have become problematic to the herbal products industry include the misidentification, mislabelling, substitution and adulteration of raw materials and finished products. With the aid of HSI and multivariate data analysis techniques, these problems can be overcome by introducing quality control procedures that allow on-line bulk analysis of samples to visually assess the chemical composition and spatial distribution of ingredients where necessary.

### 2.2. Mid-Infrared Spectroscopy Demonstrated Spectral Differences in Ginseng Root Powders

**Figure 4:**
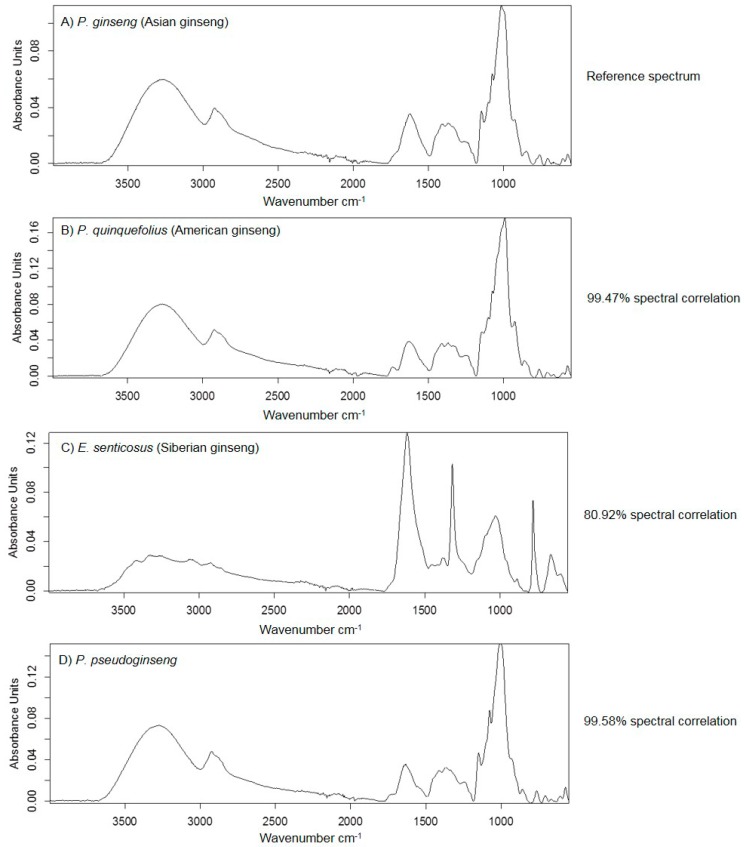
Spectral correlation of different ginseng species to *P. ginseng* “reference” material. (**A**) The reference material *Panax ginseng*; (**B**) *Panax quinquefolius*; (**C**) *Eleutherococcus senticosus* and (**D**) *Panax pseudoginseng*.

HSI is a fairly new technique that is currently under investigation in the field of natural products research and the results must be complemented using other independent techniques. To confirm the spectral differences observed using HSI, MIR was employed to analyse the spectral features of the reference materials by comparison of the spectra using the quick-compare function in OPUS**^®^**. The technique allowed for chemical correlation of each sample spectrum against the reference spectrum, *i.e.,* *P. ginseng*. The spectral correlation results (Figure 4) clearly demonstrated high spectral/chemical correlation (>99%) between *P. ginseng* (A) and *P. quinquefolius* (B) as well as *P. ginseng* (A) and *P. pseudoginseng* (D). *E. senticosus* (C) was shown to have a lower correlation (80.92%) confirming its chemical distinctness. Clear distinct signals could be observed in the fingerprint region, 1450–500 cm^−1^ for *E. senticosus* while the signal patterns for the three *Panax* species appeared similar. This is not surprising as *E. senticosus* does not belong to the *Panax* genus and thus chemotaxonomically, it would exhibit a different chemical profile when compared to other species. To use this information for product analysis, multivariate analysis techniques were applied to the MIR data of replicate samples. Orthogonal projections to latent structures-discriminant analysis (OPLS-DA) models were developed and used to predict raw material constituents in the commercial ginseng products.

**Figure 5:**
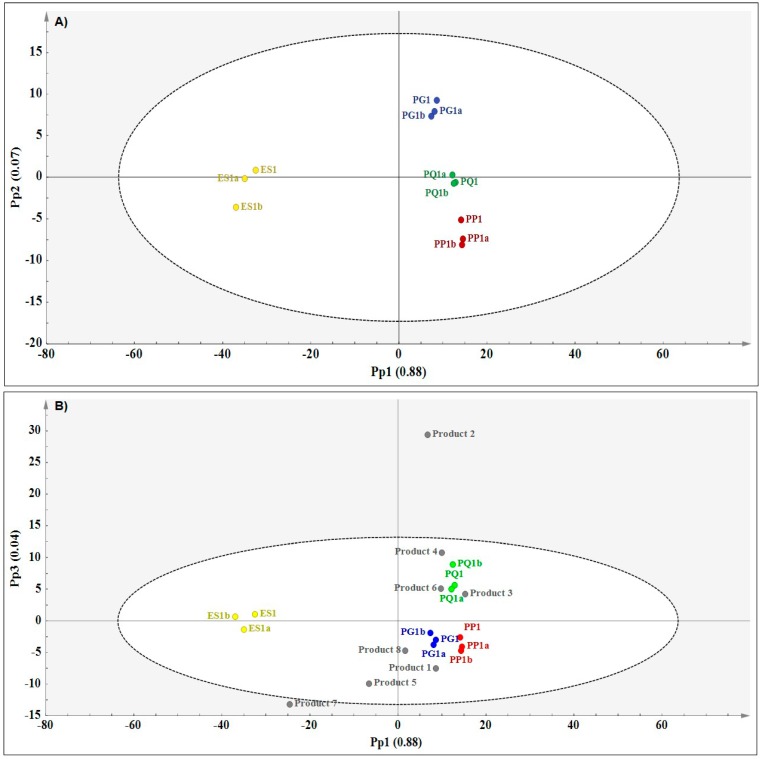
(**A**) OPLS-DA score plot (Pp1 *vs.* Pp2) showing species separation (PG—*P. ginseng* (blue); PQ—*P. quinquefolius* (green); PP—*P. pseudoginseng* (red) and ES—*E. senticosus* (yellow) and (**B**) prediction plot (Pp1 *vs.* Pp2) showing the relationships between the authentic reference materials and the commercial products based on MIR data.

The MIR data of authentic reference samples was initially analysed using PCA to observe any trends and variations within the data. As expected, some variation was observed within the data (results not shown). Following PCA, PLS-DA and OPLS-DA were applied to observe variation related to species differentiation and predict the raw materials present in each commercial product. Results of the OPLS-DA models are presented here due to ease of interpretation. Figure 5A is an OPLS-DA score plot demonstrating variation between the four ginseng reference materials. The greatest variation was observed along the first predictive component (Pp1) (88%) which clearly separated *E. senticosus* from the *Panax* species. Only 7% variation could be attributed to chemical separation of the *Panax* species along Pp2 where *P. quinquefolius* and *P. pseudoginseng* also demonstrated closer association while *P. ginseng* seemed more distinct. This correlates with the HSI results. The modelled variation responsible for separating the species using three predictive components was 98.3% (R^2^Xcum = 0.983). The model was built on pareto-scaled and SNV pre-treated data. The predictive ability of the model estimated by cross validation was 92.4% (Q^2^cum = 0.924), which demonstrates the reliability of the model in predicting future samples. Following discriminant analysis, the commercial products were introduced into the OPLS-DA model as a test set and the classification results in relation to the reference material, are presented in Figure 5B.

The proximity of the commercial products to the reference material in the score scatter plot is an indication of the likelihood of a product to contain ingredients belonging to that species. The majority of the commercial products (seven out of eight) were predicted to contain either *P. ginseng* (PG) or *P. quinquefolius* (PQ), while *E. senticosus* (ES) and *P. pseudoginseng* (PP) were unlikely raw material constituents in any of the commercial products according to MIR analysis. As can be observed from the score plot, products **1**, **5**, **7** and **8** showed close association with *P. ginseng* while products **3**, **4** and **6** were closer to *P. quinquefolius*. Although product **2** is likely to be associated with *P. quinquefolius*, its occurrence as a strong outlier outside of model boundary affects the accuracy of the prediction. To statistically assess the observed relationship between the products and the reference materials, a classification list for discriminant analysis was obtained for the data and this is illustrated in Table 2.

The table was constructed based on supervised classification of the reference materials into classes (dummy *Y*-variables). Through cross validation each sample was taken out of the model and predicted for fitness into any of the four classes as demonstrated by the predicted *Y* values (YPredPS). Cross validation results for the reference materials show that the observations are predicted well into their respective classes as YpredPS > 0.65 in all cases. To determine class membership of the commercial products, the products were introduced into the model. By matching the predicted *Y* of each product to each class (species), it is clear that products **2**, **3**, **4** and **6** were predicted as *P. quinquefolius* while products **1**, **5**, **7** and **8** were predicted as *P. ginseng* (YPredPS > 0.65). Although product 3 is predicted as *P. quinquefolius*, according to the YPredPS value the product could also be *P. ginseng* since the value (0.46) falls within the borderline range (0.35 < YPredPS < 0.65). Using MIR spectroscopy, it was possible to clearly distinguish the four ginseng reference materials and further tentatively identify raw material constituents in eight commercial products.

### 2.3. Near-Infrared Spectroscopy Demonstrated Spectral Differences in Ginseng Root Powders 

**Figure 6:**
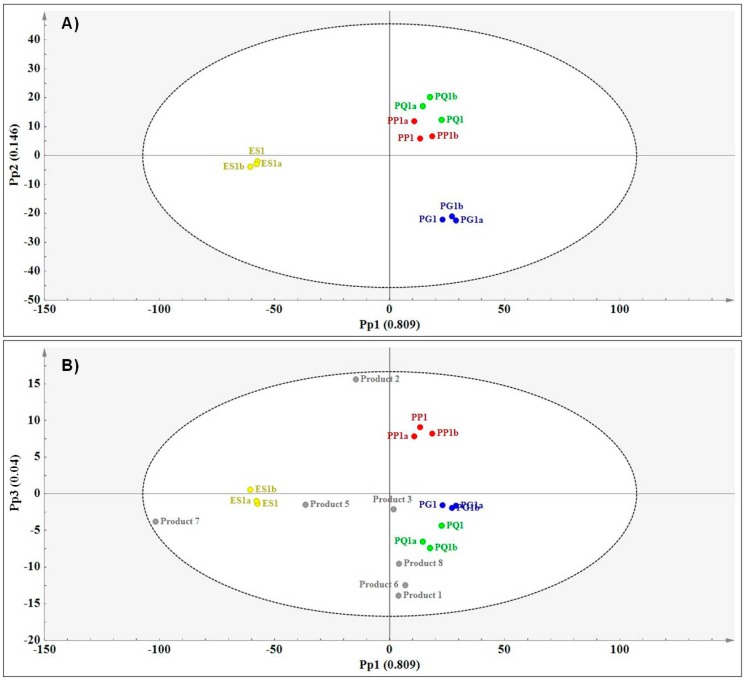
(**A**) OPLS-DA score plot (Pp1 *vs.* Pp2) showing species separation (PG—*P. ginseng* (blue); PG—*P. quinquefolius* (green); PP—*P. pseudoginseng* (red) and ES—*E. senticosus* (yellow) and (**B**) prediction plot (Pp1 *vs.* Pp3) showing the relationships between the authentic reference materials and the commercial products based on NIR data.

The usefulness of NIR spectroscopy in assessing variations among the ginseng reference materials was demonstrated. Results showed obvious differences between the species using PCA (results not shown) and OPLS-DA (Figure 6A).

The total variation modelled using a three predictive component OPLS-DA model was 99.6% (R^2^X cum = 0.996) and the predictive ability of the model was 95.3% (Q^2^cum = 0.953). As with MIR, the NIR data was pareto-scaled and SNV filtered to produce the best model statistics. Figure 6A shows the score scatter plot displaying chemical variation between the species. *E. senticosus* was clearly separated from *Panax* species along Pp1 with modelled variation of 81.0%. *Panax ginseng* clustered on Pp2−, separate from *P. quinquefolius* and *P. pseudoginseng* that clustered in close proximity to each other on Pp2+. The variation modelled along Pp2 was 14.6%. The prediction of raw material constituents based on NIR showed products **1**, **3**, **6** and **8** in close proximity to *P. quinquefolius*, product **2** close to *P. pseudoginseng* and products **5** and **7** close to *E. senticosus*. Product **4** was a strong outlier in the direction PC2+ resulting in a skewed model thus for clear visualisation of the observations, product **4** was excluded from the score plot for reporting purposes. To confirm visual assessment of the score plot, the classification list was obtained and the results are presented in Table 3.

The classification list shows that five of the eight products (**1**, **3**, **4**, **6** and **8**) were classified as either *P. quinquefolius* or *P. ginseng*. However, NIR predicts products **1** and **4** to be either *P. quinquefolius* or *P. ginseng* as shown by YPredPS > 0.65 for both products. Although the model predicts products **6** and **8** as *P. quinquefolius*, it also classifies these as borderline *P. ginseng* (YPredPS < 0.65 but > 0.35). Product **2** is predicted as *P. pseudoginseng* while products **5** and **7** are predicted as *E. senticosus*. Product **3** is predicted as borderline *P. quinquefolius*. Although some differences have been observed between NIR predictions and other spectroscopy techniques, it is clear that most products contain either *P. ginseng* or *P. quinquefolius* based on both NIR and MIR predictions. This could be due to the chemical similarities between *P. ginseng* and *P. quinquefolius*, as demonstrated using all three techniques. Thus, it was difficult for the models to conclusively classify the products as containing one of either two species and so borderline predictions were observed. Spectroscopy also suggests that *E. senticosus* is not a common ingredient in the ginseng products even though two of the products (products **3** and **6**) listed *E. senticosus* as ingredients according to the package insert. This may be a case of mislabelling or misidentification of the raw material which could translate to a consumer taking a herbal product with the hope of attaining a specific therapeutic outcome (as per label ingredient) which will not be achieved as the expected ingredient will be absent in the product. This is one of the many quality concerns in the herbal products industry and hence the need to develop robust quality control protocols based on holistic approaches. The results thus demonstrate the need for more robust, fast, holistic approaches in the analysis of herbal products to ensure standardisation and quality products that deliver the desired effects to the consumer.

### 2.4. UHPLC-MS Demonstrated Chromatographic Differences in Ginseng Root Powders

**Figure 7:**
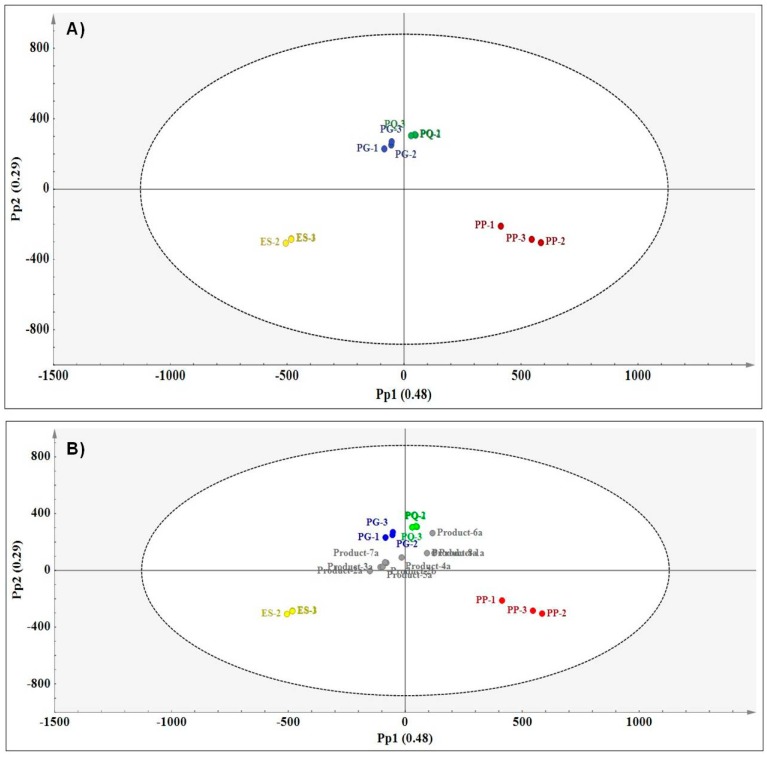
(**A**) OPLS-DA score plot (Pp1 *vs.* Pp2) showing species separation (PG—*P. ginseng* (blue); PG—*P. quinquefolius* (green); PP—*P. pseudoginseng* (red) and ES—*E. senticosus* (yellow) and (**B**) prediction plot (Pp2 *vs.* Pp2) showing the relationships between the reference materials and the commercial products based on LC-MS data.

As chromatographic techniques are commonly used in quality analyses both in industry and research, UHPLC-MS was selected to corroborate the results obtained using the three vibrational spectroscopic techniques. As with MIR and NIR, an untargeted metabolomics approach was used to analyse the full chromatographic profile. PCA and OPLS-DA models were constructed and the resulting score plots were analysed for possible chemical variation in the methanol extracts of ginseng reference materials. The results for the PCA model were not shown as they were similar to the OPLS-DA plot. The total modelled variation related to species differentiation in the OPLS-DA model was 93.8% (R^2^Xcum = 0.938) and the predictive ability of the model was estimated at 98.6% (Q^2^cum = 0.986) which shows that the model can be used for the accurate prediction of commercial products. Figure 7A is the OPLS-DA score plot showing chemical distinction of the four species where variation along Pp1 mainly separated *E. senticosus* (Pp1−) and *P. pseudoginseng* (Pp1+).

According to the chromatographic profiles of the methanol extracts, *P. ginseng* and *P. quinquefolius* share similar chemical profiles as these could not be separated along both Pp1 and Pp2. A loadings line plot was used to identify putative biomarkers (retention time/mass pairs) responsible for the chemical differences between the species clusters. Table 4 lists retention time/mass pairs and corresponding tentative identities of the compounds.

According to the OPLS-DA model, all commercial products were predicted well within the model boundary. Due to the close proximity of the commercial products to the *P. ginseng* and *P. quinquefolius* cluster, it is clear that most of the products are predicted to contain either of the two species (Figure 7B). With the aid of the classification list (Table 5), it was possible to confirm the prediction of product **6a** as *P. quinquefolius* and product **8a** as borderline *P. quinquefolius*. Five of the eight products were predicted as borderline *P. ginseng* (**1a**, **3a**, **4a**, **5a** and **7a**) and product **2a** was predicted as borderline *E. senticosus*. Using UHPLC-MS it was not possible to obtain definite predictions for most of the products as the YPredPS values for these were borderline however, it is clear that the majority of the products contain either *P. ginseng* or *P. quinquefolius*. UHPLC-MS demonstrated similar clustering patterns for both raw material differentiation and prediction of commercial products compared to the spectroscopy results. Even though UHPLC-MS is a very sensitive and accurate analytical method, it is difficult to distinguish between the *Panax* species due to the presence of many ginsenosides in almost all the species. Vibrational spectroscopy can successfully offer the same functionality at a fraction of the price.

## 3. Materials and Methods

### 3.1. Plant Material Preparation

Botanical reference materials (*P. ginseng*, *P. pseudoginseng*, *P. quinquefolius* and *E. senticosus*) with certificates of authenticity were purchased from the American Herbal Pharmacopoeia (AHP) (Scotts Valley, CA, USA). Several commercial ginseng products were purchased from local and international retailers. The formulation and product ingredient label information is provided in Table 6. The reference materials and products (removed from capsules and tea bags or crushed in the case of tablets) were ground to fine powder using a Retsch^®^ 400 MM ball mill (Haan, Germany) at a frequency of 30 Hz for 1 min. The powders were sieved through a 500 µm sieve (Endecotts Ltd., London, UK) in preparation for HSI, MIR and NIR spectroscopy. For UHPLC-MS analysis, 250 mg of each of the powdered materials were weighed and extracted with methanol (20 mL × 2) using sonication at 35 °C for 30 min. Subsequently the extracts were filtered with Whatman^®^ folded filter paper No. 41 (Munktell, Falun, Sweden). Finally, the combined filtrate was evaporated to dryness using a Genevac EZ-2 Plus Series (SP Scientific, Ipswich, UK). The dried extracts were dissolved in ultra-pure methanol (HPLC grade) to a concentration of 5 mg/mL, filtered through a 0.22 µm filter and analysed.

### 3.2. Hyperspectral Imaging (HSI) Spectroscopy

Spectral images of the powders, filled into 8 mm plastic containers, were captured in the range 920–2514 nm (spectral resolution of 6–7 nm), using a SisuCHEMA shortwave infrared (SWIR) hyperspectral imaging camera (Specim, Spectral Imaging Ltd., Oulu, Finland). The first image consisted of authentic ginseng raw material for model development and calibration while the second image comprised of eight commercial products and four authentic ginseng root powders for predictions. ChemaDAQ software (Version 3.62.183.19R) (Specim, Spectral Imaging Ltd.) was used to capture images using a high magnification (S31) lens (31 mm; 50 mm field of view; 0.3 µm spatial resolution). Images were captured at a frame rate of 100 Hz and 3.5 ms exposure time as the samples entered the field of view. Images with a pixel size of 256 × 320 and a pixel depth of 14 bits/pixel were acquired. Each sample composed of approximately 14750 pixels. Internal dark and white references were used for image calibration and to correct for variation in sample illumination.

### 3.3. Mid-Infrared (MIR) Spectroscopy Analysis

The MIR spectra of the reference material and commercial products and were recorded in the wavenumber range 4000–550 cm^−1^ on an Alpha-P Bruker FT-IR spectrometer mounted with an ATR diamond crystal (Bruker OPTIK GmbH, Ettlingen, Germany). The operating software used was OPUS**^®^** 6.5 (Bruker OPTIK GmbH). Spectra were obtained by placing a small aliquot of the powder directly onto the surface of the ATR diamond crystal. A plunger was used to press down the powder so that it was in direct contact with the crystal. A total of 32 scans were accumulated and averaged to produce an average spectrum for each sample. All measurements were performed in the absorbance mode and a spectral resolution of 4 cm^−1^ was used [15]. Chemometric analysis of the data was performed using SIMCA**^®^**-P+ 14.0 (Umetrics AB, Malmo, Sweden).

### 3.4. Near-Infrared Spectroscopy Analysis

The powdered ginseng reference and product samples were packed in Chromacol**^®^** vials (Chromacol, Sigma-Aldrich, Johannesburg, South Africa) and placed in the sample holder of a NIRFlex N500 (solids cell) FT-NIR spectrophotometer (Buchi Labortechnik AG, Flawil, Switzerland). The reflectance spectra were recorded in the wavenumber region of 10,000–4000 cm^−1^ with a spectral resolution of 4 cm^−1^ using NIRWare software (Version 1.2, Buchi Labortechnik AG, Flawil, Switzerland). A total of 32 scans were accumulated and averaged to produce a spectrum for each sample. The reflectance readings were converted to absorbance using the formula A = log (1/R) where A = Absorbance and R = reflectance, before exporting the data into SIMCA**^®^**-P+ 14.0 (Umetrics AB, Malmo, Sweden) for chemometric analyses [8].

### 3.5. Ultra-High Performance Liquid Chromatography—Mass Spectrometry Analysis

UHPLC analyses were performed on a Waters Acquity Ultra Performance Liquid Chromatographic system with PDA detector (Waters Corporation, Milford, MA, USA). To achieve chromatograms with better resolution in a short analysis time, the chromatographic conditions were optimised in a preliminary test. An Acquity UHPLC BEH C_18_ column (150 mm × 2.1 mm, i.d., 1.7 μm particle size, Waters) was used and maintained at 40 °C. The mobile phase consisted of 0.1% formic acid in water (solvent A) and acetonitrile (solvent B) at a flow rate of 0.3 mL/min; a gradient elution was as follow: 95% A: 5% B, keeping for 0.5 min, to 85% A: 15% B in 0.5 min, to 72% A: 28% B in 0.5 min, changed to 71% A: 29% B in 4 min, to 55% A: 45% B in 0.5 min, to 45% A: 55% B in 4 min, changed to 7% A: 93% B in 1 min, final changed to 100% B in 1.5 min, keeping for 1.5 min and back to initial ratio in 0.5 min. The total runtime was 16 min. The injection volume was 2.0 μL (full-loop injection). Data were collected by chromatographic software Masslynx**^®^** 4.1 (Waters Corporation, Milford, MA, USA). The UHPLC system was interfaced with a Xevo G_2_QTof mass spectrometer (Waters). The same column, elution gradient and flow rate were used during the UHPLC-MS analysis. Both positive and negative ion modes were investigated, and the results showed that higher sensitivity and more information were obtained in the negative mode. Thus, the mass spectrometer was operated in negative ion electrospray mode. N_2_ was used as the desolvation gas. The desolvation temperature was set to 350 °C at a flow rate of 600 L/h and the source temperature was 100 °C. The capillary and cone voltages were set to 2800 and 60 V, respectively. Data were collected between 100 and 1500 *m*/*z* [16]. Compound identification was performed through elementary composition to establish the possible molecular formula and comparison with literature.

### 3.6. Data Analysis

#### 3.6.1. Hyperspectral Imaging Data Analysis

The hypercubes were analysed using multivariate image analysis software (Evince**^®^** Version 2.4.0, UmBio AB, Umea, Sweden). Following conversion of reflectance values to pseudo-absorbance, PCA was performed on the data. The largest variance was observed between the background and the samples and thus image clean-up was performed by removing pixels corresponding to the background and edge effects. To improve model statistics and obtain a better visualisation of chemical differences between the species, different spectral filtering methods such as standard normal variate (SNV), multiplicative scatter correction (MSC) and Savitzky-Golay, were investigated [17]. To determine the optimum number of principal components (PCs) for the model, PCs were manually added and the Q^2^ value evaluated. The results of image analysis were presented in a score image, which displays chemical differences between species based on colour amplitude. The corresponding scatter plot indicates pixel distribution and can be coloured according to the amplitude or score values. Following PCA, image classification modelling using PLS-DA and OPLS-DA was performed by selecting the root image of each species in the PCA score image and assigning it to a class (Class 1: *P. ginseng*; Class 2: *P. pseudoginseng*; Class 3: *P. quinquefolius;* Class 4: *E. senticosus*). Full cross validation of the model was performed using the random method and the Q^2^ value was evaluated. PLS factors were added to the model and the optimum number was determined by excluding factors that did not significantly increase the value of the Q^2^cum in the model. A PLS-DA score image (Y image) was then used for class prediction of the commercial products and test set reference samples. The PLS-DA model predicts class membership by correlating pixels in the test and commercial samples to any of the four classes as per training set. The prediction table was assessed to check the pixel percentage in each image that are correlated to a specific species and these were coloured according to the predicted class in the image.

#### 3.6.2. Spectral and Chromatographic Data Analysis

Chemometric analysis of MIR, NIR and UHPLC-MS data was performed using SIMCA**^®^**-P+ 14.0 (Umetrics AB, Malmo, Sweden). The holistic analysis of UHPLC-MS chromatographic data was performed through pre-processing of the data using MarkerLynx**^®^** 4.1 software (Waters Corporation, Milford, MA, USA). Pre-processing involved peak detection, baseline correction, noise elimination and spectral alignment where molecular fragments with corresponding retention times were aligned across the whole sample set. The resulting aligned data were then listed together with the corresponding intensities across all samples and was subjected to cluster analysis in SIMCA-P**^®^**+ 14. As with HSI, PCA was initially applied to the data to investigate chemical variation between the species. Two-dimensional score plots were used as maps of observations to show the distribution of the species based on chemical differences or similarities between them. The loadings line plot was used to identify variables (retention time/mass pairs or wavenumbers) that contribute to the clustering patterns observed in the scores plot. Following PCA, PLS-DA and OPLS-DA were performed on the dataset after assigning a class to each species as previously described for PLS-DA of HSI. Based on these classification models, the botanical materials present in the commercial products were determined by class prediction of the commercial products. This was achieved by introducing the commercial samples as an external test set into the model. Using 2D prediction score plots, it was possible to visualise the predicted class of the commercial samples based on its distribution and proximity to the authentic reference sample. To statistically confirm the visual results, a classification table was obtained which showed a list of predicted *Y*-values (YPredPS) for each sample in each class/species. Class membership was defined as follows; YPredPS < 0.35; observation does not belong to the class; 0.35 < YPredPS < 0.65; observation is borderline; YPredPS > 0.65; observation belongs to the class [17].

## 4. Conclusions 

The present study demonstrates the application of HSI, MIR and NIR spectroscopies as fast, non-destructive, holistic approaches in the qualitative differentiation of ginseng raw materials. The three techniques were able to distinguish between (1) *E. senticosus* and the three *Panax* species; and (2) between *P. pseudoginseng* and *P. ginseng* together with *P. quinquefolius*. *P. ginseng* is chemically most similar to *P. quinquefolius*. These results were consistent for all three vibrational spectroscopy techniques (NIR, MIR and HSI) as well as the UHPLC-MS results. It was possible to use any of the methods to distinguish the raw material of the four different botanical species. This provides fast techniques that in addition to their non-destructive nature, allow for bulk analysis of raw material, thus saving time. The prediction models classified all the commercial products as ginseng products containing either *P. ginseng* or *P. quinquefolius* in higher proportions, as did the UHPLC-MS method. This highlights the labelling error noted for products **3** and **6**. It is evident that fast and accurate quality control protocols must be developed for botanical raw materials and commercial products.

## Figures and Tables

**Table 1:** Classification of raw material constituents in commercial ginseng products as determined from predicted pixel abundance using the PLS-DA model based on HSI data.

Product ID	HSI Prediction	Pixel Abundance (%)
P1	*P. ginseng *	99.8%
P2	*P. pseudoginseng *	87.1%
P3	*P. ginseng* & *P. pseudoginseng*	30.1% & 15.7%, respectively
P4	Not detected	-
P5	*P. ginseng *	92.3%
P6	*P. ginseng *	95.6%
P7	*E. senticosus* & *P. ginseng*	46.0% & 51.0%, respectively
P8	*P. ginseng *	97.3%

**Table 2:** Classification list showing predicted *Y*-values (YPredPS) for both the work set (reference materials) and prediction set (commercial products) based on the OPLS-DA model developed using MIR data.

YpredPS				
Sample ID	*P. quinquefolius*	*P. ginseng*	*P. pseudoginseng*	*E. senticosus*
PQ1	0.873494	0.0426901	0.0925604	−0.008744
PQ1a	0.808125	0.121958	0.0703957	−0.000479251
PQ1b	1.16977	−0.102758	−0.0755277	0.00851277
PG1	0.0139349	1.03868	−0.0411048	−0.0115117
PG1a	−0.0618089	0.978395	0.0747687	0.00864527
PG1b	0.109764	0.860837	−0.00149277	0.0308912
PP1	0.132295	0.0800257	0.800855	−0.0131765
PP1a	0.00459008	−0.0117866	1.01447	−0.00727496
PP1b	−0.0494571	−0.0360678	1.08239	0.00313148
ES1	0.0991252	0.105236	−0.139046	0.934686
ES1a	−0.139707	0.122022	0.0264342	0.991251
ES1b	0.0398709	−0.199232	0.0952921	1.06407
Product **1**	−0.412435	1.36797	0.0813636	−0.036902
Product **2**	3.0505	−1.75931	−0.552881	0.261688
Product **3**	0.742292	0.436145	−0.0797367	−0.0987007
Product **4**	1.35676	−1.00639	0.488739	0.160889
Product **5**	−0.758334	1.58308	−0.0816218	0.256879
Product **6**	0.789701	0.279934	−0.100268	0.0306327
Product **7**	−1.21907	2.03352	−0.400351	0.585898
Product **8**	−0.217563	1.52262	−0.384549	0.0794879

PQ—*P. quinquefolius*; PG—*P. ginseng*; PP—*P. pseudoginseng*; ES—*E. senticosus*. YPredPS < 0.35–does not belong to the class; 0.35 < YPredPS < 0.65–borderline; YPredPS > 0.65–belongs to the class.

**Table 3:** Classification list showing predicted Y-values (YPredPS) for both the work set (reference materials) and prediction set (commercial products) based the OPLS-DA model developed using NIR data.

YpredPS				
Sample ID	*P. quinquefolius*	*P. ginseng*	*P. pseudoginseng*	*E. senticosus*
PQ1	0.813187	0.122776	0.117013	−0.0529759
PQ1a	0.995349	−0.0142832	−0.0259548	0.0448885
PQ1b	1.11345	−0.0642308	−0.0497072	0.00048305
PG1	−0.0101584	0.972106	−0.0092678	0.0473198
PG1a	0.00959447	1.01391	0.00216237	−0.0256659
PG1b	0.0448257	0.975509	−0.0134489	−0.00688598
PP1	−0.0725259	0.0364964	1.01859	0.0174423
PP1a	0.102231	−0.113137	0.976149	0.0347581
PP1b	0.0116467	0.0560867	0.979388	−0.0471219
ES1	0.0611244	−0.0011869	−0.0428528	0.982915
ES1a	0.020713	0.0148458	−0.0231686	0.98761
ES1b	−0.0894418	0.00110899	0.0710999	1.01723
Product **1**	0.84396	0.700825	−0.848236	0.303451
Product **2**	−0.508698	−0.251922	1.43319	0.32743
Product **3**	0.378945	0.281583	0.10016	0.239313
Product **4**	4.47139	1.40538	−5.08383	0.207057
Product **5**	0.223186	0.0177814	0.0501989	0.708833
Product **6**	0.898712	0.535932	−0.673811	0.239166
Product **7**	−0.37724	0.307427	−0.54973	1.61954
Product **8**	0.773699	0.421224	−0.445524	0.2506

PQ—*P. quinquefolius*; PG—*P. ginseng*; PP—*P. pseudoginseng*; ES—*E. senticosus*. YPredPS < 0.35–does not belong to the class; 0.35 < YPredPS < 0.65–borderline; YPredPS > 0.65–belongs to the class.

**Table 4:** Putative biomarkers (retention time/mass pairs) and tentative identification of compounds responsible for the chemical differences between the observed clusters based on UHPLC-MS data.

Retention Time (min)	*m*/*z* Ratio	Tentative Identification
***E. senticosus* Cluster**		
2.8395	191.048; 353.088; 707.183	Chlorogenic acid
3.2841	191.056; 515.12	3′,5′-*O*-dicaffeoylquinic acid
12.1911	277.217; 295.228	Panaxytriol
***P. pseudoginseng* Cluster**		
3.4817	931.526; 932.529	Notoginsenoside R1
7.8212	945.542; 946.546	Ginsenoside Rd
7.1892	1119.590	
7.4358	769.473	Notoginsenoside R2
7.5839 7.6484	783.488 683.436	Ginsenoside Rg2 Ginsenoside Rh1
***P. ginseng* and *P. quinquefolius* Cluster**		
7.3329	799.484; 800.487	Ginsenoside Rf (F11)
7.4753	956.493; 955.49	Ginsenoside Ro
7.8768	793.437	Chikusetsusaponin

**Table 5:** Classification list showing predicted Y-values (YPredPS) for both the work set (reference materials) and prediction set (commercial products) based on the OPLS-DA model developed using UHPLC-MS data.

YPredPS				
Sample ID	*P. quinquefolius*	*P. ginseng*	*P. pseudoginseng*	*E. senticosus*
PQ-1	1.00046	−0.00238603	0.00992292	−0.00799578
PQ-2	1.01809	−0.0189166	0.00451271	−0.00368392
PQ-3	0.97584	0.0189336	−0.00432262	0.00954945
PG-1	−0.0197475	0.980594	0.00182605	0.0373279
PG-2	0.0282955	0.964586	0.0117777	−0.004659
PG-3	−0.010917	1.04486	−0.00000587	−0.0339342
PP-1	0.0490762	0.0607009	0.841733	0.0484899
PP-2	−0.027449	−0.0330929	1.08988	−0.0293363
PP-3	−0.0115143	−0.0140102	1.03378	−0.00825432
ES-1	0.00570655	0.0102809	0.00376177	0.980251
ES-2	−0.00811097	−0.0207769	0.000141397	1.02875
ES-3	0.000274241	0.00923075	0.0069962	0.983499
Product-**1a**	0.174703	0.559364	0.281116	−0.015183
Product-**2a**	0.176647	0.328216	0.105444	0.389693
Product-**3a**	0.156203	0.400995	0.130551	0.312251
Product-**4a**	0.290633	0.376308	0.159322	0.173737
Product-**5a**	0.155012	0.410041	0.13663	0.298317
Product-**6a**	0.717904	0.220726	0.130851	−0.0694814
Product-**7a**	0.176875	0.442111	0.121741	0.259273
Product-**8a**	0.453183	0.249427	0.234023	0.0633671

PQ—*P. quinquefolius*; PG—*P. ginseng*; PP—*P. pseudoginseng*; ES—*E. senticosus*. YPredPS < 0.35—does not belong to the class; 0.35 < YPredPS < 0.65—borderline; YPredPS > 0.65—belongs to the class.

**Table 6:** Formulation and labelling information for the commercial ginseng products purchased.

Sample ID	Type	Type of Formulation	Active Ingredients
1	Korean ginseng (*Panax ginseng*)	Capsules	Standardised Korean ginseng (*Panax*) 250 mg Root powdered extract (20 mg [8%] ginsenosides) Raw Korean ginseng root powder 200 mg
2	Ginseng (Energy and vigor)	Tablets	*Panax ginseng* 100 mg
3	Ginseng (Herbal health)	Tablets	Korean ginseng (*P. ginseng* root) 66.66 mg Siberian ginseng (*E. senticosus* root) 50 mg Indian ginseng (Ashwaganda root) 34 mg
4	American ginseng	Tablets	American ginseng, mannitol, corn starch, magnesium stearate.
5	Ginseng (Organic herb and flower infusion)	Tea bags	Lemongrass, peppermint, rose hip, orange peel, liquorice, cardamom, ginseng root, cinnamon, ginseng flowers, ginger, citrus extract, nettle, alfalfa, black pepper, horsetail, celery seeds, cloves, dried kombucha drink.
6	Ginseng root (*Eleutherococcus ginsengwurzel*)	Tea bags	*E. senticosus* 50 g
7	Pure ginseng slimming tea	Tea bags	Oolong tea, lotus leaves, senna leaves, cassia seeds (semen cassia), ginseng and fructus momordicae.
8	*Panax ginseng*	Tea bags	*Panax ginseng* root

## Abbreviations

The following abbreviations are used in this manuscript:

AHP	American Herbal Pharmacopoeia
CNS	central nervous system
HPLC-ELSD	high performance liquid chromatography coupled to evaporative light scattering detection
HPLC-UV	high performance liquid chromatography couple to ultraviolet detection
HSI	hyperspectral imaging
LC-MS	liquid chromatography
MIR	mid-infrared spectroscopy
MVA	multivariate analysis
NIR	near infrared spectroscopy
NMR	nuclear magnetic resonance
(O)PLS-DA	(orthogonal) partial least squares discriminant analysis
PC	principal component
PCA	principal component analysis
PLS	partial least squares
SNV	standard normal variate
TLC	thin layer chromatography
UHPLC-MS	ultra high performance liquid chromatography coupled to mass spectrometry
YPredPS	predicted Y values
